# Mode of Action of Lactoperoxidase as Related to Its Antimicrobial Activity: A Review

**DOI:** 10.1155/2014/517164

**Published:** 2014-09-16

**Authors:** F. Bafort, O. Parisi, J.-P. Perraudin, M. H. Jijakli

**Affiliations:** ^1^Plant Pathology Laboratory, Liége University, Gembloux Agro-Bio Tech, Passage des Déportés 2, 5030 Gembloux, Belgium; ^2^Taradon Laboratory, Avenue Léon Champagne 2, 1480 Tubize, Belgium

## Abstract

Lactoperoxidase is a member of the family of the mammalian heme peroxidases which have a broad spectrum of activity. Their best known effect is their antimicrobial activity that arouses much interest in *in vivo* and *in vitro* applications. In this context, the proper use of lactoperoxidase needs a good understanding of its mode of action, of the factors that favor or limit its activity, and of the features and properties of the active molecules. The first part of this review describes briefly the classification of mammalian peroxidases and their role in the human immune system and in host cell damage. The second part summarizes present knowledge on the mode of action of lactoperoxidase, with special focus on the characteristics to be taken into account for *in vitro* or *in vivo* antimicrobial use. The last part looks upon the characteristics of the active molecule produced by lactoperoxidase in the presence of thiocyanate and/or iodide with implication(s) on its antimicrobial activity.

## 1. Introduction

Mammalian peroxidases are distinct from plant peroxidases in size, amino acid homologies, nature of the prosthetic group, and binding of the prosthetic group to the protein. Plant peroxidases consist of approximately 300 amino acids with a noncovalently bound heme moiety, while mammalian peroxidases have 576 to 738 amino acids with a covalently bound heme moiety [1]. Animals' peroxidases display high sequence homology compared to plant peroxidases [2, 3]. Mammalian peroxidases can detoxify peroxide, protect against pathogens, and induce the production of thyroid hormones, while plant peroxidases trigger defense reactions against pathogens and stress, remove hydrogen peroxide, and are involved in the metabolism of lignin and auxin and in the oxidation of toxic reductors [1].

Based on amino acids homologies, peroxidases are now classified into two superfamilies. The first superfamily clusters peroxidases from plant, archea bacteria, and fungi and is classified into three classes. Class I is composed of intracellular peroxidases such as yeast cytochrome *c* peroxidase, ascorbate peroxidase, and catalase peroxidase, Class II is formed by secretory fungal peroxidases such as manganese and lignin peroxidases, and Class III consists in secretory plant peroxidases including one of the most studied peroxidases, the horseradish peroxidase [4, 5]. The second one is called the peroxidase-cyclooxygenase superfamily and clusters mammalian peroxidases together with protein from invertebrate, bacterial, plant, and fungal species and other metalloproteins such as cyclooxygenase [5]. This latter superfamily originally called “the myeloperoxidase family” shares a domain of about 500 amino acids corresponding to the catalytic site [6] and is classified into several subfamilies; one of them is the chordata peroxidases in which the mammalian peroxidases are found [5]. The main clades are the myeloperoxidases (MPO), eosinophil peroxidases (EPO), and the lactoperoxidase (LPO) branch. Another clade consists in thyroid peroxidase (TPO) which is distantly related to MPO, EPO, and LPO [5].

MPO is a lysosomal constituent in neutrophils and macrophages and displays antimicrobial activity during the postinfection inflammatory process but is also involved in acute inflammatory diseases and in other pathologies such as atherosclerosis [7–10]. EPO is secreted in eosinophils and displays cytotoxic activity against parasites, bacteria, and fungi. It could be associated to allergic eosinophilic inflammatory disease pathologies [1, 10, 11]. LPO and salivary peroxidase are found in secretions of exocrine glands and are associated to antibacterial and antifungal activity [12–14]. Finally, TPO is a membrane enzyme localized in thyroid follicle cells that takes part in the synthesis of thyroid hormones [1].

This review summarizes present knowledge on the mode of action of lactoperoxidase which can be extended to the mammalian peroxidases mode of action and on the specific interaction of LPO with thiocyanate and iodide, together or alone, with implications on its antimicrobial activity.

## 2. Mode of Action of Lactoperoxidase

LPO is a calcium- and iron-containing glycoprotein arranged in a single polypeptide chain of about 80 kDa [15, 18, 19]. Human LPO is moderately cationic with a pI of* ca*. 7.5 but bovine LPO is more cationic with a pI of* ca*. 9.6 [18, 19]. MPO is a highly cationic (pI of* ca.* 10) dimeric protein of 146 kDa, each monomer containing one calcium and iron and joined together by a disulfide bridge [18]. The ion calcium plays an important role in the stability of both enzymes [19, 20]. The active site in LPO, MPO, EPO, and TPO is the heme which consists in a protoporphyrin IX derivative fixed covalently through two ester linkages via a conserved distal aspartate and glutamate residues, a third link being present in MPO and consisting in a thioether sulfonium bond with a methionine [4, 15, 19, 21]. These covalent bonds, which are specific for vertebrate peroxidases, result in a distortion of the symmetry and planarity of the prosthetic group and give the unique spectroscopic and redox properties of these proteins [4, 20, 21]. The proximal heme ligand is a highly conserved histidine residue which is hydrogen bonded to a conserved asparagine residue that acts as hydrogen-bond acceptor [20, 22]. On the distal heme site, a conserved histidine-arginine couple plays a role in the proton transfer during the formation of Compound I, and a conserved glutamine residue and several conserved water molecules are involved in a hydrogen-bond network acting in halide delivery and binding [4, 18, 20]. A conserved asparagine residue in LPO, EPO, and MPO located close to the distal histidine seems also critical for the catalysis mechanism [18, 20]. The substrate channel in LPO is narrower, longer, and more hydrophobic compared to MPO with the consequence that the LPO-active site seems to be less exposed to surrounding media [22, 23].

**Figure 1:**
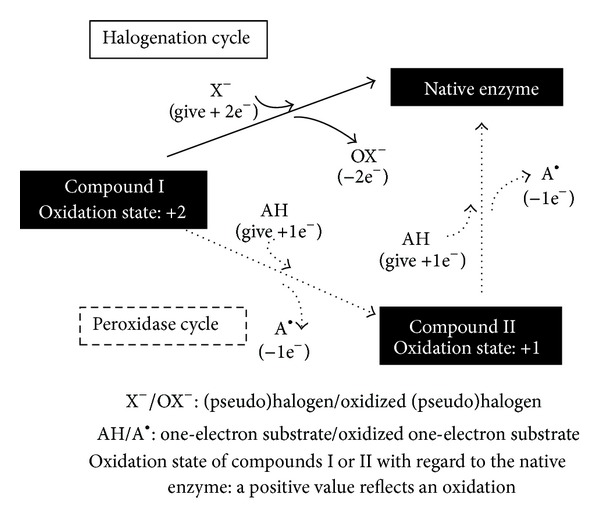
Halogenation or peroxidase cycle of peroxidases Compound I.

Heme peroxidases are oxidoreductase enzymes that act through different reaction mechanisms. Although some characteristics are specific to a member of the family, the same global procedure is followed by all members. The cycle begins with the transformation of the native enzyme into Compound I. Afterwards, and depending mainly on substrate concentrations, Compound I enters the halogenation cycle or the peroxidase cycle which both end by the enzyme returning to its native state (see Figure 1).

### 2.1. Formation of Compound I

The first reaction of the native enzyme starts in the presence of hydrogen peroxide, which acts as a relatively specific electron acceptor [24]. Other substrates have been described, such as ethyl hydroperoxide, peroxyacetic acid, cumene hydroperoxide, and 3-chloroperoxybenzoic acid [20, 25]. Compound I is formed as follows:

(1) $$\begin{array}{c} \text{Peroxidase}\;\left(\text{native}\;\text{form}\right)+{\text{H}}_{2}{\text{O}}_{2}⟶\text{Compound}\;\text{I}+{\text{H}}_{2}\text{O} \end{array}$$

The native enzyme undergoes a two-electron oxidation. Two electrons are transferred from the enzyme to hydrogen peroxide which is reduced into water. Compound I is two oxidizing equivalents above the native enzyme: one is in the oxyferryl heme center and the other is present as an organic cation located on the porphyrin ring [4, 26, 27]. Compound I is not specific regarding the electron donor [24] and the composition of the medium determines its subsequent cycle (see Figure 1). As Compound I is very unstable, an amino acid residue of the apoprotein is oxidized in the absence of an exogenous electron donor [14]. This reaction yields Compound I isomer that is very similar to Compound II regarding its iron redox state and its incapability to react with halogens [20].

### 2.2. The Halogenation Cycle

In the presence of a halogen (Cl^−^, Br^−^, or I^−^) or a pseudohalogen (SCN^−^), Compound I is reduced back to its native enzymatic form through a two-electron transfer while the (pseudo)halogen is oxidized into a hypo(pseudo)halide (see Figure 1). Hypo(pseudo)halides (OX^−^) are powerful oxidants with antimicrobial activity [14, 28–32]. The halogenation cycle is described by the following reaction:

(2) Compound  I+X−  (halogen  or  pseudo-halogen) ⟶Native  enzyme+OX−

**Figure 2:**
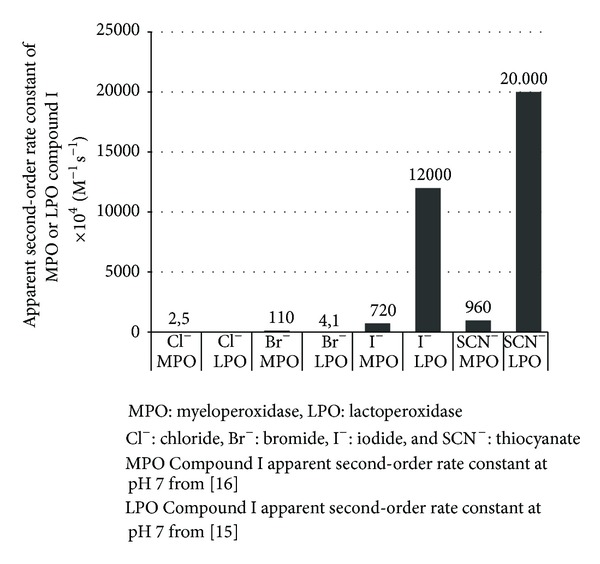
Apparent second-order rate constant at pH 7 (×10^4^ M^−1^ s^−1^) of the reaction between myeloperoxidase Compound I or lactoperoxidase Compound I with (pseudo)halides [15, 16].

The oxidation rate of halogens by peroxidase-derived Compound I depends on various factors. One of these factors is the standard reduction potential of the enzyme, which differs among peroxidases and plays a role in their capacity to oxidize specific (pseudo)halides. The redox reaction can occur only if the reduction potential of the enzyme is equal or superior to the reduction potential of the substrate. The standard reduction potential at pH 7 of Compound I peroxidases and the couple of two-electron reduction HOX/X^−^ is ranking in the following ascending rank: LPO Compound I < EPO Compound I < MPO Compound I; HOSCN/SCN^−^ < HOI/I^−^ ≪ HOBr/Br^−^ < HOCl^−^/Cl^−^ [17, 18, 33, 34]. This involves that only the MPO Compound I is able to oxidize Cl^−^ with appropriate rates, LPO being able to oxidize with high rates I^−^ and SCN^−^ and slowly Br^−^ [16, 17, 20, 33, 34]. Interestingly, although LPO Compound I has the lowest reduction potential compared to EPO Compound I and MPO Compound I, it catalyzes the oxidation of I^−^ and SCN^−^ with the highest rates (Figure 2) [15–17, 20]. This suggests that other factors play a role, such as anion size, anion access, and anion binding as well as structural and amino acid differences in the active and binding site between enzymes [17, 18, 20].

**Figure 3:**
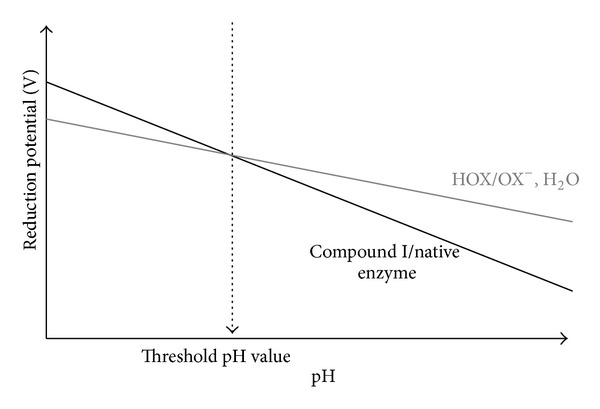
Illustration (according to [17]) of the pH threshold value above which the oxidation of the halogen by mammalian heme peroxidase will be thermodically unfavorable. The reduction potential of the couple Compound I/native enzyme and the couple halogen (X = chloride, bromide) HOX/OX^−^ is expressed with an illustrative function of the pH, at a specific concentration of enzyme and substrates.

The reduction potential of the Compound I/native enzyme and HOX/X^−^ redox couples depends on reactant concentrations and pH values. At a specific reactant concentration, it decreases with increasing pH values, but slopes differ (Figure 3) [17]. This means that there exists a threshold pH value above which the oxidation of halides becomes thermodynamically unfavorable, especially for halides with high reduction potential such as Cl^−^ and Br^−^ [17].

**Figure 4:**
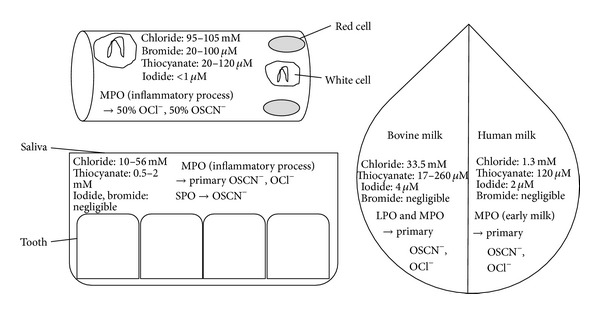
Illustration of the interaction between the biodisponibility of a peroxidase, the (pseudo)halogen concentration in plasma, in saliva, and in milk, and the production of oxidant molecules. MPO: myeloperoxidase; SPO: salivary peroxidase; LPO: bovine lactoperoxidase; OCl^−^: hypochlorite; and OSCN^−^: hypothiocyanite. Although chloride is the most available substrate compared to thiocyanate, bromide, and iodide, thiocyanate is the most effective substrate for the Compound I and hypothiocyanite could be produced at equal or superior levels compared to hypohalides.

Concentrations of (pseudo)halogens also affect their affinity to Compound I (Figure 4).

Although plasma Cl^−^ concentrations are 1,000-fold higher than Br^−^ and SCN^−^, MPO Compound I oxidizes similar amounts of SCN^−^ and Cl^−^ [35]. EPO Compound I preferentially oxidizes SCN^−^ in the presence of physiological concentrations of SCN^−^, Br^−^, and I^−^ [36]. In the saliva of healthy adults, where Cl^−^ concentrations are only about 25-fold higher than SCN^−^, SPO Compound I and MPO Compound I primarily generate hypothiocyanite [37, 38]. The levels of I^−^ in human milk, saliva, blood, and tissues except the thyroid gland are below 1 *μ*M and its* in vivo* oxidation by Compound I is negligible [17, 38]. In human milk, peroxidase activity is only derived from leucocytes. As MPO is able to oxidize Cl^−^ and Cl^−^ milk concentration is high, oxidation of Cl^−^ is possible although it has never been reported [14]. In bovine milk, lactoperoxidase is an abundant enzyme, and with mean concentrations of I^−^ and SCN^−^ of 310 *μ*g/kg and 0.2–15 mg/kg, respectively, oxidation is possible [19, 39]. Nevertheless, the relative abundance of SCN^−^ in all secretions, blood, and tissues and its better capacity as an electron donor make it one of the main* in vivo* substrates of Compound I lactoperoxidase and myeloperoxidase for 2-electron oxidation compared to halides [15]. In* in vitro* applications, the ratio between (pseudo)halides regulates the ratio of hypohalides generated by the reaction. However, as SCN^−^ is the most effective substrate for Compound I, its presence, even in small quantities, enhances its oxidation [14, 35, 36].

### 2.3. The Peroxidase Cycle

Alternatively, Compound I can shift to the peroxidase cycle, which consists of two sequential one-electron transfers back to the enzyme that yield (i) Compound II and (ii) the native enzyme, while the substrate is oxidized into a radical (Figure 1) [40–42]. The peroxidase cycle is summarized in the following equations:

(3) $$\begin{array}{c} \text{Compound}\;\text{I}+\text{AH}⟶\text{Compound}\;\text{II}+{\text{A}}^{∙}\\ \text{Compound}\;\text{II}+\text{AH}⟶\text{Native}\;\text{enzyme}+{\text{A}}^{∙} \end{array}$$

Compound I is not specific regarding the one-electron donor; it can be exogenous or endogenous, and a lot of candidates have been described [18, 20, 43]. Hydrogen peroxide can undergo a one-electron oxidation only with MPO Compound I, with the formation of superoxide [16, 20, 44, 45].

During the first step of the peroxidase cycle, the cation located in the porphyrin ring undergoes a one-electron reduction with formation of Compound II and concomitant oxidizing of one one-electron substrate [4, 24]. Compound II maintains one oxidizing equivalent in the oxyferryl center [4, 24]. Finally, this latter is reduced back to the native enzyme with the oxidation of a second one-electron donor.

The standard reduction potential of the couple Compound I/Compound II is high and allowed the one-electron oxidation by Compound I of a wide range of substrates [18, 20]. In contrast, the standard reduction potential of the couple Compound II/native enzyme is low and restrains the numbers of possible substrates for Compound II [18, 20]. With the result that (i) the Compound II/native enzyme standard reduction potential is too low to react with halogens and (ii) the nature of substrates strongly influenced their ability to be oxidized by mammalian peroxidase compound II [2, 14, 20], therefore, when the enzyme is in this state, it has to be first reduced to the ground state before possibly participating to the halogenation cycle and producing antimicrobial molecules [14]. Moreover, the reduction of Compound II to the ground state is the rate-limiting step [45, 46]; that is, the peroxidase cycle interferes with the halogenation cycle and slows down antimicrobial activity [47].

The peroxidase cycle has been described as a possible catalytic sink for nitric oxide (NO) [46] but also for hydrogen peroxide in the case of a moderate excess of H_2_O_2_ relative to LPO [24]. Increase of NO removal from media, even in presence of Cl^−^, after addition of MPO, EPO, or LPO and accelerated rates of Compound I and Compound II reduction in presence of NO show that peroxidases may regulate the bioavailability of NO [46]. In conditions of high excess of hydrogen peroxide relative to LPO and in the absence of an exogenous electron donor, Compound II is transformed into Compound III which is 3 oxidative equivalents above the native enzyme. In moderate excess conditions, Compound III can be partially reconverted into Compound II and can reenter the peroxidase cycle [24, 40]. Otherwise, the enzyme is irreversibly inactivated; the heme fraction is cleaved and iron is released [48]. In the presence of an exogenous two-electron donor, the enzyme is largely protected from hydrogen peroxide because the halogenation cycle is favored. Furthermore, protection is higher with iodide because oxidized iodide consumes H_2_O_2_ to produce oxygen and iodide in a reaction called the pseudocatalytic activity of peroxidase [24, 40, 49].

However, thiocyanate can act as a one-electron donor and be part of the peroxidase cycle, with the sequential formation of two thiocyanate radicals [47]. With 200 *μ*M SCN^−^, LPO is predominantly in its native form; this indicates that the halogenation cycle prevails [47].

In the presence of both one- and two-electron donors, competition for oxidation can occur and favor the halogenation or the peroxidase cycle. The presence of EDTA inhibits the oxidation of iodide due to competition for binding to Compound I [50]. The standard reduction potential between the donors favors the molecule with the lowest reduction potential. Thereby, the respective reduction potentials of the one- and two-electron oxidation of thiocyanate at very low pH are 1.65 V and 0.82 V and promote the halogenation cycle [51]. In the case of low concentrations of halides or thiocyanate, below 10 *μ*M I^−^ or 3 *μ*M SCN^−^, Compound I reacts with any suitable exogenous or endogenous one-electron donor, with the subsequent formation of Compound II and a negligible oxidation rate of halides and thiocyanate [14].

### 2.4. Inhibition of the Function of Mammalian Heme Peroxidase

The function of heme peroxidases can be inhibited in several ways that could be classified into three categories. The first one could represent an inhibition of the enzyme by (i) molecules or proteins and (ii) external conditions such as pH and temperature. For example, cyanide, azide, nitrite, mercaptomethylimidazole, thiourea, superoxide, high levels of nitric oxide, and high levels of thiocyanate bind to the native enzyme and alter Compound I formation [20, 46, 47, 52–54]. With thiocyanate, inhibition is linked to the restriction of the binding site to hydrogen peroxide and the interaction of SCN^−^ with a water molecule [23]. High concentration of H_2_O_2_ or I^−^ will inactivate irreversibly LPO with liberation of free iron [48, 55]. Temperature between 73°C and 83°C, depending on the heating time, results in unfolding and inactivation of LPO [19]. Extreme pH is inactivating enzymes and at low pH an amino acid group, probably histidine, is protonated which prevents the binding of H_2_O_2_ [56]. Some proteases such as pepsin and pronase are able to inactivate LPO by proteolysis but chymotrypsin did it very slowly and trypsin and thermolysin are not active against LPO [19].

The second group of inhibitors could concern substances or proteins which are able to interfere with the catalytic mechanism. For example, catalase consumes H_2_O_2_ and will stop the formation of Compound I [30, 52]. Competition between substrates can also interfere with the reaction cycle such as SCN^−^ which competes very effectively with Cl^−^, Br^−^, and I^−^ [52, 53]. HOCl has the capacity to bind to LPO native enzyme and convert it into Compound I. Above 100 *μ*M, HOCl mediates the destruction of the LPO heme center [57].

The third class could be related to substances or proteins which are buffering active molecules produced during the catalytic reaction. For example, presence of thiosulfate, thioglycolate, glutathione, dithiothreitol, cysteine, NAD(P)H, and tyrosine will reduce the antimicrobial activity through reacting with OCl^−^, OBr^−^, OI^−^, or OSCN^−^ [52, 53, 58, 59]. The enzyme NADH-OSCN oxidoreductase is able to reduce OSCN^−^ in SCN^−^ [60].

## 3. Activity of Lactoperoxidase with Thiocyanate and/or Iodide 

LPO concentrations in cow's milk are around 30 mg L^−1^ depending on season, diet, and calving and breeding season [61]. LPO extraction from whey or milk is based on a well-developed industrial process [62]. Compared to MPO and EPO, LPO is easily isolated and manufactured in large quantities. As a result, cow's milk peroxidase is the favorite molecule for* in vitro* or* in vivo* applications such as conservation of raw and pasteurized milk, storage of emulsions and cosmetics, moisturizing gel and toothpaste in human dry mouth, veterinary products, and preservation of foodstuffs [19, 61, 63, 64].

### 3.1. Activity of LPO Related to Hypothiocyanite

#### 3.1.1. Mode of Action of Hypothiocyanite

Thiocyanate is oxidized in a two-electron reaction that yields hypothiocyanite. Hypothiocyanite has a p*K*a of 5.3 [65]. It is more acidic than hypohalides that have p*K*as of 7.5 (HOCl), 8.6 (HOBr), and 10.6 (HOI) [14, 66]. All hypo(pseudo)halides (OX^−^) are in an acid-base equilibrium association with their corresponding acid hypo(pseudo)halide (HOX). For example, in the case of hypothiocyanite,

(4) $$\begin{array}{c} \text{HOSCN}⇆{\text{OSCN}}^{-}+{\text{H}}^{+} \end{array}$$

The acid form has a higher oxidation potential and is more soluble in nonpolar media so that it passes through hydrophobic barriers such as cell membranes more easily but it is less stable than the basic form (OX^−^) [14, 66]. Hypohalide acids are predominant in acidic to neutral media and even in basic conditions for HOBr and HOI, whereas hypothiocyanite needs a pH below 5.3 to be predominant in the acid form [66, 67].

**Figure 5:**
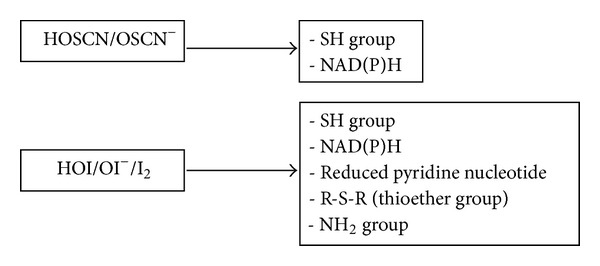
Target group of hypothiocyanite, hypoiodite, and iodine. Due to its low oxidation power, hypothiocyanite is relatively specific and is not reactive against all thiols.* In vivo*, hypoiodite seems to be selectively directed against reduced pyridine nucleotide because even the presence of excess glutathione and methionine does not thoroughly inhibit their oxidation. HOSCN/OSCN^−^: acidic or basic form of hypothiocyanite; HOI/OI^−^: acidic or basic form of hypoiodite; and I_2_: iodine.

SCN^−^ is the two-electron donor with the lowest reduction potential and therefore forms the hypothiocyanite acid with the lowest oxidative power compared to hypohalous acids. Hypohalous acids rank as follows, with increasing oxidative strength: OSCN^−^ < OI^−^ < OBr^−^ < OCl^−^ [28, 66]. These characteristics make hypothiocyanite relatively specific regarding its molecular target (Figure 5), that is, a thiol moiety [28, 59, 68].

Sulfhydryl oxidation by OSCN^−^ generates sulfenyl thiocyanate, in equilibrium with sulfenic acid [68]:

(5) $$\begin{array}{c} {\text{SCN}}^{-}+{\text{H}}_{2}{\text{O}}_{2}+\text{LPO}⟶{\text{OSCN}}^{-}+\text{LPO}\\ \text{R-SH}+{\text{OSCN}}^{-}⟶\text{R-S-SCN}+{\text{OH}}^{-}\\ \text{R-S-SCN}+{\text{H}}_{2}\text{O}⟶\text{R-S-OH}+{\text{SCN}}^{-}+{\text{H}}^{+} \end{array}$$

**Figure 6:**
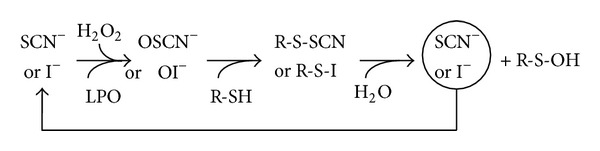
Illustration of the cofactor role of SCN^−^ or I^−^. When the necessary conditions are fulfilled, that is, (i) no substrate competitor for SCN^−^ or I^−^ for binding to lactoperoxidase, (ii) enough peroxidase, H_2_O_2_ and SCN^−^ or I^−^, (iii) enough R-SH, and (iv) no incorporation of SCN^−^ or I^−^ in stable byproducts, the quantity of OSCN^−^ or OI^−^ produced depends only on the amount of H_2_O_2_. SCN^−^: thiocyanate; I^−^: iodide; H_2_O_2_: hydrogen peroxide; LPO: lactoperoxidase; R-SH: peptide or protein with a thiol moiety; R-S-SCN or R-S-I sulfenyl thiocyanate or iodide; R-SOH: sulfenic acid; OSCN^−^: hypothiocyanite; and OI^−^: hypoiodite.

The cycle of reactions shows that thiocyanate acts like a cofactor for LPO (Figure 6) so that the total number of oxidized sulfhydryls is independent of SCN^−^ as long as (i) thiocyanate is not exhausted, (ii) thiocyanate is not in competition with other substrates for the binding to Compound I, (iii) thiocyanate is not incorporated into an aromatic amino acid, (iv) enough H_2_O_2_ is present, and (v) thiol moiety is still available [68, 69].

Although the target of OSCN^−^ is a thiol moiety, not all sulfhydryls are equally sensitive to OSCN^−^; albumin, cysteine, mercaptoethanol, dithiothreitol, glutathione, and 5-thio-2-nitrobenzoic acid are all oxidized but *β*-lactoglobulin is poorly oxidized, probably due to a limited accessibility of sulfhydryls to OSCN^−^ [68]. In some conditions, that is, the joint presence of LPO, enough H_2_O_2_ and SCN^−^, and after the oxidation of available sulfhydryls, modification of tyrosine, tryptophan, and histidine protein residues can occur and that could be linked to the formation of a labile powerful oxidant such as sulfur dicyanide [68].

Some authors suggest that (SCN)_2_ is formed during the enzymatic reaction and then chemically hydrolyzed into hypothiocyanite [14, 69, 70]. However, a recent publication demonstrates that (SCN)_2_ cannot be a precursor during the enzymatic oxidation of SCN^−^ at neutral pH in mammals [71].

Hypothiocyanite is less stable in acid conditions, with high concentrations of SCN^−^ and in the presence of (SCN)_2_, and it is thought to break down* via* the following net reaction [14]:

(6) $$\begin{array}{c} 4\text{HOSCN}+{\text{H}}_{2}\text{O}⟶3{\text{SCN}}^{-}+{\text{CNO}}^{-}+{{\text{SO}}_{4}}^{2-}+6{\text{H}}^{+} \end{array}$$

A recent study, based notably on spectroscopic and chromatographic methods, proposes the following net equation within the 4–7 pH range:

(7) 3HOSCN+H2O⟶XSO42−+XHCN+(1−X)SO32−+(1−X)CNO−+2SCN−+(5−X)H+

The proportions of end anions were different at pH 4 and pH 7; at pH 7, the proportion of CNO^−^ was higher, SCN^−^ formation was slower, and no CN^−^ was detected [71].

It might seem easier to produce hypothiocyanite chemically in* in vitro* applications, but producing hypothiocyanite chemically from the oxidation of SCN^−^ by a halogen (Cl_2_ or Br_2_) or by a hypohalous acid (HOCl or HOBr) in basic media is tricky due to overoxidation of SCN^−^ [66]. The reference method in the literature to produce 1- to 2-day stable OSCN^−^ is by hydrolyzing (SCN)_2_ in basic conditions [72–74].

Hypothiocyanite inhibitors have been described. For example CN^−^, a weak acid buffer, dissolved carbonate, excess hydrogen peroxide, hydrofluoric acid, metallic ions, glycerol, or ammonium sulfate accelerates the decomposition of OSCN^−^, whereas sulfonamide stabilizes it [67, 72].

Appropriate concentrations of substrates induce enhanced activity [75].

#### 3.1.2. Biological Activity of Hypothiocyanite

**Figure 7:**
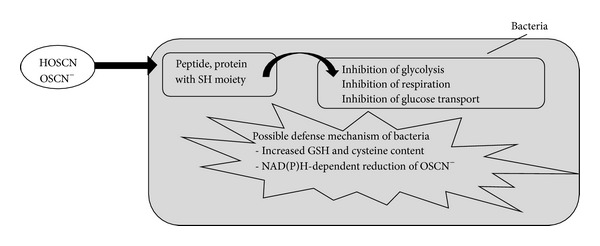
Biological activity of hypothiocyanite on bacteria and possible defense mechanism of the bacteria. Reversible inhibition is observed in that (i) hypothiocyanite is not reactive against all thiols and (ii) if hypothiocyanite is removed or diluted, the pathogen recovers. Irreversible inhibition is linked to (i) long period of incubation, (ii) the bacterial species, and (iii) hypothiocyanite concentration. HOSCN/OSCN^−^: acidic or basic form of hypothiocyanite and GSH: glutathione.

The biological activity of hypothiocyanite is summarized in Figure 7.

The sulfhydryl moiety is essential for the activity of numerous enzymes and proteins. Inhibition of bacterial glycolysis through the oxidation of hexokinase, glyceraldehyde-3-phosphate dehydrogenase (GAPDH), aldolase, and glucose-6-phosphate dehydrogenase has been observed [14, 51, 65, 70, 76]. Inhibition of respiration and glucose transport is associated with the alteration of cell membranes or transporters [14, 51, 65, 77]. Irreversible inhibition is linked to long periods of incubation and bacterial sensitivity depends on the bacterial species and on hypothiocyanite concentrations [14, 51, 59]. Increased concentrations of reducing agents such as glutathione and cysteine can reverse the inhibition through buffering hypothiocyanite and converting the reduced thiol back into sulfhydryl [14, 78]. This defense mechanism is used by* Escherichia coli*; it induces the CysJ promoter during the stress response to the lactoperoxidase system [79]. Another resistance mechanism could be the NAD(P)H-dependent reduction of OSCN^−^ without any loss of the sulfhydryl compound [14, 72, 78]. Alteration of the bacterial membrane increases the efficacy of hypothiocyanite [80].

Furthermore, the activity of the entire system (enzyme + substrates) is known to be more effective than hypothiocyanite alone, whether enzymatically or chemically produced. This has been explained by the production of short-lived, highly reactive intermediates such as O_2_SCN^−^ and O_3_SCN^−^ by the enzyme or by the oxidation of OSCN^−^ in conditions of excess H_2_O_2_ [65, 73, 81]. The activity of hypothiocyanite has been described against bacteria such as* Actinomyces spp.*,* Bacillus cereus*,* Lactobacillus spp*.,* Staphylococcus albus*,* S. aureus*,* Streptococcus spp*.,* Escherichia coli*,* Legionella pneumophila*,* Salmonella typhimurium*,* Pseudomonas fluorescens*,* P. aeruginosa*,* Campylobacter jejuni*,* C. coli,* and* Listeria monocytogenes* [14, 32]. Reversible inhibition is observed when cells recover after OSCN^−^ is depleted [14, 59]. Irreversible inhibition is obtained with long-term incubation and high level of OSCN^−^ [59]. Higher concentration of SCN^−^ compared to I^−^ is necessary to obtain inhibition against* E. coli* and accumulation of OSCN^−^ is observed as it is not reactive against all thiols [59]. Therefore, the activity of the SCN^−^-LPO system appears to be more bacteriostatic than bactericidal.

### 3.2. Activity of LPO Related to Oxidized Iodide

#### 3.2.1. Chemistry of Oxidized Iodide

Iodide is oxidized by Compound I through a single two-electron transfer that yields oxidized I^−^ in the form of I_2_ or HOI [14, 24, 82–85]. The active agent is composed of a mixture of species that are not yet formally detailed due to the very complex behavior and stability of I_2_ and HOI in aqueous environments that strongly depend on pH values and iodide concentrations [66, 82, 83, 86].

**Figure 8:**
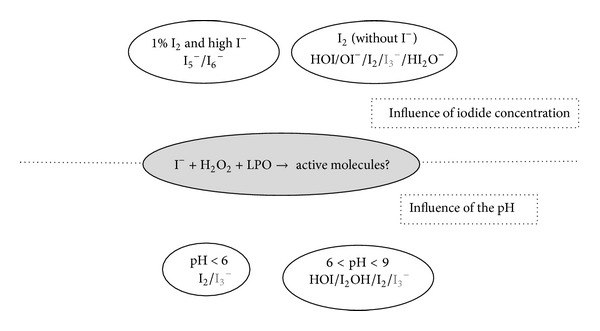
Illustration of the molecules that can be present after oxidation of iodide by lactoperoxidase in presence of H_2_O_2_. The active species depend mainly on the concentration of iodide (upper part) and the pH (lower part). The species with an oxidant power are represented in bold.

Based on the inorganic chemistry of iodine in water and literature on enzymatic oxidation of iodide, the active molecules have been described as follows (Figure 8).

(i) Under pH 6 and in the presence of iodide, only I_2_, I^−^, and I_3_^−^ are present and the only active molecule is I_2_. I_2_ concentrations decrease with increasing concentrations of I^−^. At an initial 1 mM I_2_, with I^−^ concentrations ranging from 1 mM to 100 mM, I_2_ concentrations fall from almost 1 mM to 0,01 mM, as described by the following association reaction [24, 82, 83, 86]:
  (8) $$\begin{array}{c} {\text{I}}_{2}+{\text{I}}^{-}⇆{{\text{I}}_{3}}^{-} \end{array}$$
(ii) In solution within a 6–9 pH range and with a maximum 1 mM iodide, a mixture of HOI/I_2_OH/I_2_/I_3_^−^ is formed in which I_3_^−^ is not active and I_2_OH is probably less reactive than HOI or I_2_ [86, 87]. If I^−^ concentrations are above 10 mM, I_3_^−^ represents the main species formed and the concentration of active molecules relatively drops. The mechanism is summarized in the following net equations:
  (9) HOI+I−+H+⇆I2OH−+H+⇆I2+H2O⇆I2+I−⇆I3−
(iii) In iodine solution, without iodide or when available iodide has been oxidized, the number of I_2_-derived molecules decreases with decreasing I_2_ concentrations. At 1,000 *μ*M I_2_, with pH-related ratios, five relevant species are observed (I_2_, HOI, I_3_^−^, HI_2_O^−^, and OI^−^). At 10 *μ*M I_2_, the main species are only I_2_, HOI, and OI^−^, and HOI could represent up to 90% of the active oxidant molecules at pH 8-9 [86]. Below a pH of 10.6, the following reactions are involved:
  (10) $$\begin{array}{c} {\text{I}}_{2}+{\text{H}}_{2}\text{O}⇆\text{HOI}+{\text{I}}^{-}+{\text{H}}^{+}\left(\text{hydrolysis}\;\text{of}\;{\text{I}}_{2}\right)\\ {\text{I}}_{2}+{\text{I}}^{-}⇆{{\text{I}}_{3}}^{-}\left(\text{triiodide}\;\text{formation},\text{independent}\;\text{of}\;\text{pH}\right) \end{array}$$
(iv) At high I^−^ and 1% I_2_ concentrations as in Lugol solution, I_5_^−^ and I_6_^−^ are formed and represent 8.2% of the active oxidative agents [86], after the following reaction:
  (11) $$\begin{array}{c} {{\text{I}}_{3}}^{-}+{\text{I}}_{2}⇆{{\text{I}}_{5}}^{-}\left(\text{pentaiodide}\;\text{formation}\right)\\ 2{{\text{I}}_{3}}^{-}⇆{{\text{I}}_{6}}^{2-}\;\left(\text{dimerization}\;\text{of}\;{{\text{I}}_{3}}^{-}\right) \end{array}$$

The stability of HOI and I_2_ is linked to their disproportionation in iodate, which has no oxidative activity in neutral and basic pH conditions [86]. The disproportionation reactions read as follows:

(12) $$\begin{array}{c} 3\text{HOI}⇆{{\text{IO}}_{3}}^{-}+2{\text{I}}^{-}+3{\text{H}}^{+}\;\left(\text{disproportionation}\;\text{of}\;\text{HOI}\right)\\ 3{\text{I}}_{2}⇆{{\text{IO}}_{3}}^{-}+5{\text{I}}^{-}+6{\text{H}}^{+}\;\left(\text{disproportionation}\;\text{of}\;{\text{I}}_{2}\right) \end{array}$$

I_2_ stability increases at higher pH values and higher iodide concentrations [86]. In drinking water, HOI disproportionation is slow and varies substantially; HOI has a half-life of 4 days to 3.5 years depending on (i) the initial level of HOI that speeds its decomposition and (ii) the presence of borate, phosphate, or carbonate that catalyzes its decomposition [88, 89].

#### 3.2.2. Mode of Action of Oxidized Iodide

The oxidative strength of I_2_ is between that of the corresponding hypohalous acid HOI and the hypoiodite ion OI^−^ and ranks as follows: 0.485 V (OI^−^) < 0.536 V (I_2_) < 0.987 V (HOI) [66].

HOI reacts through very rapid oxidation of thiol groups, oxidation of NAD(P)H, oxidation of *β*-nicotinamide mononucleotide, direct reaction with thioether groups through sulfoxidation, and slow oxidation of the amine moiety (Figure 5) [87, 90, 91]. At low I^−^ concentrations, iodination of tyrosine residues is catalyzed by the enzyme [14]. In a cellular environment, HOI seems to be more selectively directed against the degradation of reduced pyridine nucleotides than HOCL and HOBr because even the presence of excess glutathione, methionine, or oxidized glutathione does not thoroughly inhibit their oxidation [87].

In some conditions, that is, (i) enough iodide, H_2_O_2_, and peroxidase, (ii) no accumulation of oxidized iodide, and (iii) no incorporation of iodide into stable byproducts such as tyrosine residues, iodide acts as a cofactor (Figure 6) and the proportion of oxidized sulfhydryls is proportional to the amount of H_2_O_2_ as described below [85, 92]:

(13) 2I−+H2O2+LPO  (native  enzyme) ⟶I2+2H2O+LPO  (native  enzyme)R-SH+I2⟶R-S-I+I−+H+R-S-I+H2O⟶R-S-OH+I−+H+

In the case of high concentrations of I^−^ and/or H_2_O_2_, inhibition of tyrosine iodation has been observed [83] and related to the pseudocatalytic redox degradation of H_2_O_2_ with formation of O_2_ when excessive H_2_O_2_ is present (reaction 1) and production of I_3_^−^ when excessive amounts of I^−^ are present (reaction 2):

(14) $$\begin{array}{c} {\text{I}}_{2}+{\text{H}}_{2}\text{O}⟶{\text{O}}_{2}+2{\text{I}}^{-}+2{\text{H}}^{+}\left(\text{reaction}\;1\right)\\ {\text{I}}_{2}+{\text{I}}^{-}⇆{{\text{I}}_{3}}^{-}\left(\text{reaction}\;2\right) \end{array}$$

Both reactions deplete the amount of the active oxidizing agent I_2_. In the absence of tyrosine, oxidized iodide reacts with nucleophilic molecules such as I^−^, Cl^−^, or OH^−^ to form I_2_, I_3_^−^, ICl, ICl_2_, IOH, and I_2_OH [82]. Some anions such as Cl^−^, HPO_4_^−^, or OH^−^ reduce the amount of I_2_/I_3_^−^ but this effect is inversely proportional to the concentration of I^−^; above pH 9, I_2_ is hydrolyzed and IO_3_^−^ is formed [82].

HOI can be produced chemically through oxidation of I^−^ by Cl_2_ or O_3_, with a short half-life due to overoxidation of HOI by Cl_2_ and O_3_ [89] and through oxidation of I^−^ by HOCl, HOBr, or NH_2_Cl with a longer half-life [87, 89].

#### 3.2.3. Biological Action of Oxidized Iodide

**Figure 9:**
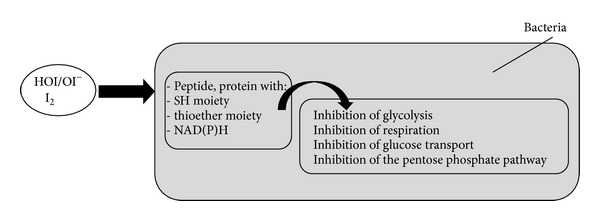
Biological activity of hypoiodite or iodine on bacteria. Irreversible inhibition is observed and could be linked to (i) oxidation of thiol groups, NAD(P)H, and thioether groups, (ii) high reactivity of HOI/I_2_ against thiol and reduced nicotinamide nucleotides, and (iii) the incorporation of iodide in tyrosine residue of protein (iodination of protein). HOI/OI^−^: acid or basic form of hypoiodite and I_2_: iodine.

The biological action of oxidized iodide (Figure 9) is similar to that of hypothiocyanite but differs in that (i) the reactivity of oxidized iodide is complete against thiol group and (ii) cells did not recover after removing of oxidized iodide [59].

Due to the cofactor role of I^−^, inhibition of respiration in* Escherichia coli* in the presence of LPO, H_2_O_2_, and I^−^ is complete with only 10 *μ*M NaI, whereas 100 *μ*M of solely I_2_ is necessary to obtain complete inhibition. This is directly related to the oxidation of sulfhydryls, not to the percentage of iodine incorporation [92, 93].

*E. coli* seems to be more sensitive if the bacteria are incubated together with the entire system (enzyme, H_2_O_2_, and iodide) rather than adding several minutes after mixing the enzyme with its substrates. This could be linked to the formation of an unstable reactive intermediate [52].

The activity of the I^−^ peroxidase system is more effective against* E. coli* than the SCN^−^ system, in that lower I^−^ concentrations are necessary, all sulfhydryls are oxidized, and cells do not recover even if the amount of I_2_ is not sufficient to oxidize all SH groups [59, 80]. Against* L. acidophilus*, high non physiological amounts of I^−^ are necessary to obtain inhibition whereas small concentrations of SCN^−^ are effective [70].

CN^−^, azide, EDTA, and SCN^−^ inhibit the formation of oxidized iodide [50, 52]. Increased pH values and increased amounts of thiol and NAD(P)H compounds reduce the activity of the iodide peroxidase system [52].

LPO-H_2_O_2_-I^−^ in presence of* Streptococcus mitis* is active against* Staphylococcus aureus* and* E. coli* [94]. LPO-H_2_O_2_-I^−^ is active against* Micrococcus*,* S. aureus*,* Listeria monocytogenes*,* Bacillus cereus*,* E. coli,* and* Candida albicans* [12, 19, 80]. In the presence of other peroxidases, the I^−^ peroxidase system is active against* Schistosoma mansoni*,* Fusarium nucleatum,* and* Actinobacillus actinomycetemcomitans* [31, 95, 96]. Compared to SCN^−^, I^−^-LPO shows bactericidal activities [14, 19, 80].

### 3.3. Activity of LPO Related to Hypoiodite and Hypothiocyanite

The combination of SCN^−^ with I^−^ in the lactoperoxidase system has been poorly studied. Tackling the enzymatic mechanism is tricky, and contradictory results have been found about microbial activity in the concomitant presence of SCN^−^ and I^−^.

In the presence of SCN^−^ and I^−^, there is competition between the two substrates for oxidation by lactoperoxidase [14, 36]. I^−^ alone exhibits bactericidal activity, but an SCN^−^/I^−^ ratio of 0.1 inhibits that bactericidal effect, and an SCN^−^/I^−^ ratio of 1 antagonizes it due to competition for oxidation and faster decomposition of HOSCN in the presence of I^−^ [14]. Against* A*.* actinomycetemcomitans*, the peroxidase system with I^−^, Cl^−^, or a combination of I^−^ and Cl^−^ is effective but addition of SCN^−^ cancels the antibacterial effect [96]. On the other hand, a synergistic or unaffected effect of iodide in the SCN^−^-H_2_O_2_-LPO system has been shown against* Candida albicans*,* E. coli*,* S. aureus*,* Aspergillus niger,* and* Pseudomonas aeruginosa* [19, 97].

## 4. Conclusion

The molecular evolution of heme peroxidases and the preservation of their catalytic domain [6] show that the production of strong oxidants is a powerful part of the nonimmune defense mechanisms against pathogenic bacteria, fungi, or parasite which made the use of those enzymes in practical applications worthwhile.

The enzymatic reactions involving mammalian peroxidases are complex and various molecules can promote or reduce dramatically the antibacterial activity of the peroxidase system. In order to favor the halogenation cycle required in* in vitro* and* in vivo* antimicrobial applications, several points have to be taken into account: (i) to avoid the presence of competitors to iodide or thiocyanate for binding to Compound I and to avoid the presence of inhibitors of the enzyme or of active molecules, (ii) to avoid excess H_2_O_2_ concentration which is able to destruct the enzyme and to react with iodine or hypoiodite with loosing of active molecules, (iii) to favor the presence of hypoiodite instead of iodine due to the association reaction of iodine with iodide, (iv) to avoid excess concentration of thiocyanate which can inhibit formation of Compound I, (v) to use the entire system (enzyme + substrates) instead of active molecules alone, (vi) to favor moderate acid pH when hypothiocyanite is the active molecule, (vii) for bactericidal, fungicidal, or parasitical applications, the use of iodide has to be preferred, (viii) the use of combined presence of iodide and thiocyanate has to be checked carefully for efficacy, and (ix) to favor the cofactor role of iodide or thiocyanate.
